# Sex Differences in the Evaluation of Congestion Markers in Patients with Acute Heart Failure

**DOI:** 10.3390/jcdd9030067

**Published:** 2022-02-24

**Authors:** Pietro Scicchitano, Claudio Paolillo, Micaela De Palo, Angela Potenza, Silvia Abruzzese, Marco Basile, Antonia Cannito, Maria Tangorra, Piero Guida, Pasquale Caldarola, Marco Matteo Ciccone, Francesco Massari

**Affiliations:** 1 Cardiology Section, Hospital “F. Perinei”, 70022 Bari, Italy; pot.angela@gmail.com (A.P.); silvia.abruzzese@libero.it (S.A.); dr.marcobasile@gmail.com (M.B.); antonia.cannito77@gmail.com (A.C.); maradoct@libero.it (M.T.); franco_massari@libero.it (F.M.); 2 Cardiology Section, Hospital “Umberto I”, 70033 Bari, Italy; claudio.paolillo68@gmail.com; 3 Cardiac Surgery Unit, Azienda Ospedaliero-Universitaria Policlinico Bari, 70124 Bari, Italy; micaela.depalo85@gmail.com; 4 Cardiology Section, Hospital “Miulli”, Acquaviva delle Fonti, 70021 Bari, Italy; pieroguida@libero.it; 5 Cardiology Section, S. Paolo Hospital, 70132 Bari, Italy; pascald1506@gmail.com; 6 Section of Cardiovascular Diseases, Department of Emergency and Organ Transplantation, University of Bari, 70124 Bari, Italy; marcomatteo.ciccone@uniba.it

**Keywords:** sex, congestion, acute heart failure, plasma volume status, BUN/Cr ratio

## Abstract

The impact of sex on the assessment of congestion in acute heart failure (AHF) is still a matter of debate. The objective of this analysis was to evaluate sex differences in the evaluation of congestion at admission in patients hospitalized for AHF. We consecutively enrolled 494 AHF patients (252 female). Clinical congestion assessment, B-type natriuretic peptide levels analysis, blood urea nitrogen to creatinine ratio (BUN/Cr), plasma volume status estimate (by means of Duarte or Kaplam-Hakim PVS), and hydration status evaluation through bioimpedance analysis were performed. There was no difference in medications between men and women. Women were older (79 ± 9 yrs vs. 77 ± 10 yrs, *p* = 0.005), and had higher left ventricular ejection fraction (45 ± 11% vs. 38 ± 11%, *p* < 0.001), and lower creatinine clearance (42 ± 25 mL/min vs. 47 ± 26 mL/min, *p* = 0.04). The prevalence of peripheral oedema, orthopnoea, and jugular venous distention were not significantly different between women and men. BUN/Cr (27 ± 9 vs. 23 ± 13, *p* = 0.04) and plasma volume were higher in women than men (Duarte PVS: 6.0 ± 1.5 dL/g vs. 5.1 ± 1.5 dL/g, *p* < 0.001; Kaplam–Hakim PVS: 7.9 ± 13% vs. −7.3 ± 12%, *p* < 0.001). At multivariate logistic regression analysis, female sex was independently associated with BUN/Cr and PVS. Female sex was independently associated with subclinical biomarkers of congestion such as BUN/Cr and PVS in patients with AHF. A sex-guided approach to the correct evaluation of patients with AHF might become the cornerstone for the correct management of these patients.

## 1. Introduction

Sex difference is a well-established issue in a heart failure (HF) setting [1,2] as well as in the context of acute myocardial infarction [3]. Women with HF effectively show advanced age as compared to men, non-ischemic aetiology of HF, higher incidences of HF with preserved ejection fraction (HFpEF), and more symptomatic forms of HF [4,5,6,7,8].

Congestion is one of the main features of HF [9]. The evaluation of congestion plays a central role in the general management of patients with acute heart failure (AHF), thus improving diagnosis, providing prognostic information, and guiding treatments [10]. 

The higher the number of signs and symptoms of congestion, the higher the risk of 30-day mortality rate in patients with AHF (up to 156% in cases of the presence of 6–7 symptoms/signs) [11]. The literature provides data about the prognostic impact of signs of congestion in patients with both acute and chronic HF [12,13,14]. The absence of congestion is tightly related to improvements in the overall survival of patients with HF, even in those with New York Heart Association (NYHA) class IV [15,16].

Nevertheless, the literature is scant regarding the influence of sex on congestion markers in patients with HF, and conflicting data emerge from the analysis of the studies [17,18,19,20,21]. Data from the CHARM-Preserved (candesartan in heart failure: assessment of the reduction in mortality and morbidity) (EF ≥ 45%), I-Preserve (irbesartan in heart failure with preserved ejection fraction), and TOPCAT-Americas (treatment of preserved cardiac function heart failure with an aldosterone antagonist trial) effectively outlined higher prevalence in signs and symptoms of congestion in women than men with HFpEF [18]. A subanalysis of the ‘Placebo-controlled Randomized Study of the Selective A1 Adenosine Receptor Antagonist Rolofylline for Patients Hospitalized with Acute Decompensated Heart Failure and Volume Overload to Assess Treatment Effect on Congestion and Renal FuncTion’ (PROTECT) did not report significant differences between men and women according to signs and symptoms of congestion in patients with HF [20]. Indeed, randomized controlled trials (RCTs) did not consider further and novel biomarkers of congestion such as plasma volume status (PVS), bioimpendace vector analysis (BIVA), and/or blood urea nitrogen to creatinine (BUN/Cr) ratio and determinants of congestion biomarkers such as serum colloid osmotic pressure (COP) and serum osmolality (Osm) in addition to physical examination.

The aim of this research was to investigate the influence of sex on congestion status of patients with AHF.

## 2. Materials and Methods

### 2.1. Study Populations

This was a retrospective study. Patients with AHF admitted to the Cardiology Unit of Altamura Hospital, Bari (Italy), between January 2010 and November 2013, were enrolled. We collected data related to physical and clinical characteristics, comorbidities, laboratory evaluations, arterial blood gas parameters, and pharmacological treatments upon the admission to the intensive care unit.

All patients underwent echocardiographic assessment in order to evaluate left ventricular ejection fraction (LVEF). Simpson’s method was adopted. Patients were defined in relation to LVEF into three groups according to international guidelines: “reduced LVEF” (HFrEF, LVEF <40%), “mildly reduced LVEF” (HFmrEF, LVEF between 40 and 49%), and “preserved LVEF” (HFpEF, LVEF ≥50%) [22].

The laboratory and instrumental evaluations were performed as routine measurements upon admission to our intensive care unit.

Patients younger than 18 years, with acute coronary syndrome, recent cardiac surgery intervention, and/or life-threatening malignancy were excluded.

The study complied with the Declaration of Helsinki and was approved by the local Institutional Review Board. Written informed consent was obtained from each patient at inclusion (protocol n. 0081801/CE—29 October 2015, study number: 4816).

### 2.2. Estimation of COP, Osmolality and Glomerular Filtration Rate 

Total serum proteins (TP) were measured using the Biuret method (normal range: 6.6–8.3 g/dL) and albumin (A) using the immune-turbidimetry method (normal range: 3.5–5.2 g/dL). Serum globulin concentration (“G”) derived by the difference between TP and A (G = TP − A). COP (mmHg) was calculated using the formula of Landis–Pappenheimer: COP = A/TP × (2.8 × TP + 0.18 × TP^2^ + 0.012 × TP^3^) + G/TP × (1.6 × TP + 0.15 × TP^2^ + 0.006 × TP^3^). COP normal values were 25 ± 2 mmHg [23]. Serum osmolality (mOsm/kg) was calculated using the following formula: [2 × Sodium (mmol/L)] + [BUN (mg/dL)/2.8] + [Glucose (mg/dL)/18] [24]. The estimated glomerular filtration rate (eGFR) was calculated using the modification of diet in renal disease (MDRD) equation: 186.3 × Cr^−1.154^ × (age in years)^−0.203^ × 1.212 (if patient was black) × 0.742 (if patient was female) [25]. 

### 2.3. Brain Natriuretic Peptide

Brain natriuretic peptide (BNP) levels were measured at admission using a microparticle enzyme immunoassay (Architect, Abbott Park, IL, USA). The intra- and inter-assay variability coefficients ranged from 0.9% to 5.6% and 1.7% to 6.7%, respectively.

### 2.4. BUN to Creatinine Ratio

BUN and serum creatinine were measured with a Beckman Coulter AU 680 chemistry analyser. The BUN/Cr median value in the general population is 15.0 (interquartile range (IQR): 12.9–17.6) [26].

### 2.5. Estimated Plasma Volume Status (PVS)

The PVS was calculated by means of both Duarte’s and Kaplan–Hakim’s formulas. Duarte’s formula was calculated as follows: Duarte-PVS (in dL/g): [(100 − haematocrit (%)/haemoglobin (g/dL)] [27].

Estimated plasma volume, as assessed by Kaplan–Hakim formula (KH-PVS), was calculated by comparing actual plasma volume (aPVS) to ideal plasma volume (iPVS) [28,29]. The aPV was calculated as follows: aPVS = (1 − haematocrit) × [a + (b × body weight in kg)] (a = 1530 in males and a = 864 in females, b = 41.0 in males and b = 47.9 in females), iPVS = c × body weight in kg (c = 39 in males and c = 40 in females), and relative PVS = [(actual plasma volume -ideal plasma volume)/(ideal plasma volume] × 100 (%)]. Relative PVS represents the mean percentage deviation of patients from their ideal plasma volume.

### 2.6. Bioimpedance Vector Analysis

BIVA was assessed at admission on the right side of the body using tetrapolar impedance plethysmography that emitted 50 kHz alternating sinusoidal current (CardioEFG, Akern RJL Systems, Florence, Italy) [30,31]. The two vector components R and Xc of BIVA were recorded and divided by the subject’s height. (R/Xc graph). The results were visualized as a BIVA-derived hydration percentage (hydration index, %). This value was calculated by an equation that used two components, R and Xc (Bodygram 1.4, Akern RJL Systems, Florence, Italy). Normal values are in the range between 72.7% and 74.3%, corresponding to the 50th percentile of the R/Xc graph [30].

### 2.7. Hydra Score

The HYDRA score is a simple score which we previously validated [14]. Briefly, the HYDRA score is based on four congestion biomarkers: BNP >441 pg/mL, BUN/Cr >25, Duarte-PVS >5.3 dL/g, and Hydration index (as assessed by BIVA) >73.8 %. The combination of each component of this “congestion” score improves the prognostic evaluation of patients with HF [14].

### 2.8. Statistical Analysis

Continuous variables were reported as means ± standard deviation. Categorical variables are expressed as counts (percentages) or medians with 95% confidence intervals. The comparisons between groups were performed using the Student’s *t*-test and Chi-squared tests for continuous variables and categorical variables, respectively. A one-way ANOVA was used to analyse the differences between means. If we obtained significant ANOVA results, we adopted the post-hoc test of Student–Newman–Keuls in order to explore the mean differences among pairs of groups.

Multivariate analyses were performed by logistic regression to analyse the factors associated with sex at univariate analysis. Odds ratios (ORs) with 95% confidence intervals (CIs) were given. *p*-values below 0.05 were defined as statistically significant. The analyses were performed using STATA software, version 12 (StataCorp, College Station, TX, USA).

## 3. Results

Four-hundred and ninety-four patients with AHF were included in this study: 252 women and 242 men. Table 1 summarizes the characteristics of the two groups (male and female).

No significant differences were found according to comorbidity prevalence, body mass index (BMI), serum electrolytes, creatinine levels, serum COP, serum osmolality, or arterial blood gas parameters. Men showed higher prevalence in pacemaker/implantable cardioverter defibrillator (27% vs. 15%, *p* = 0.001). Women demonstrated a higher prevalence in HFpEF as compared to men (51% vs. 26%, *p* < 0.001), while the prevalence of HFrEF was higher in men than women (64% vs. 39%, *p* < 0.001).

We tried to evaluate the impact of the type of HF (HFrEF, HFmrEF, and HFpEF) on the congestion markers in relation to sex. Table 2 summarizes the main results.

Only BNP was different among the three groups as both men and women with HFrEF showed higher values in BNP as compared to those with HFmrEF and HFpEF. No differences were according to other congestion parameters (Table 2).

**Figure 1:**
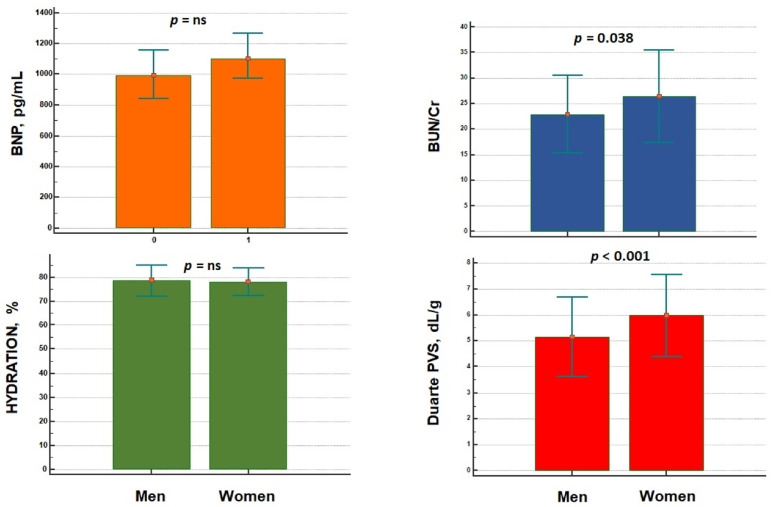
Differences in congestion biomarkers between male and female patients with acute heart failure. **BNP:** brain natriuretic peptide; **BUN/Cr ratio:** blood urea nitrogen/creatinine ratio; **PVS:** plasma volume status.

**Figure 2:**
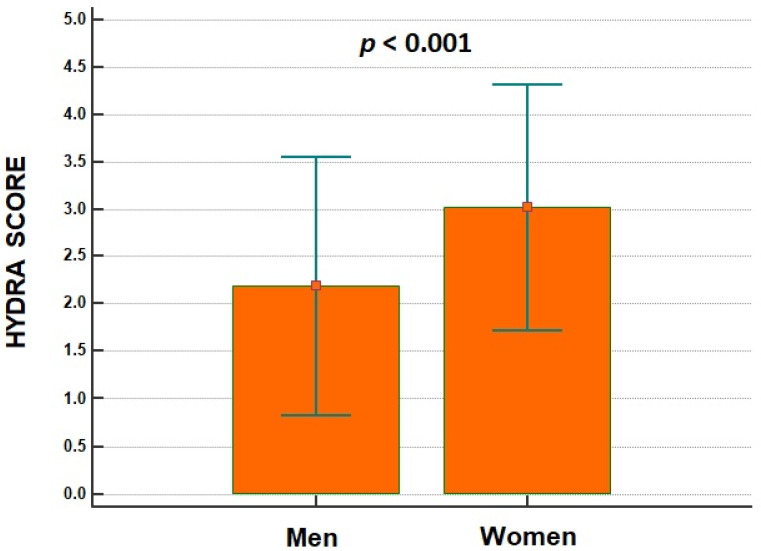
Different behaviour of HYDRA score between women and men admitted for acute heart failure.

Women were also older than men (79 ± 9 years vs. 77 ± 10 years, *p* = 0.005), and had higher plasma concentration in BUN (38 ± 20 mg/dL vs. 36 ± 19 mg/dL, *p* = 0.019) and lower eGFR values than men (42 ± 25 mL/min/1.73 m^2^ vs. 47 ± 26 mL/min/1.73 m^2^, *p* = 0.039). Physical examination did not report any differences in terms of peripheral oedema, orthopnoea and jugular venous distention. BUN/Cr ratio and Duarte PVS were significantly higher in women than men (Figure 1), as well as Hydra score (Figure 2).

**Figure 3:**
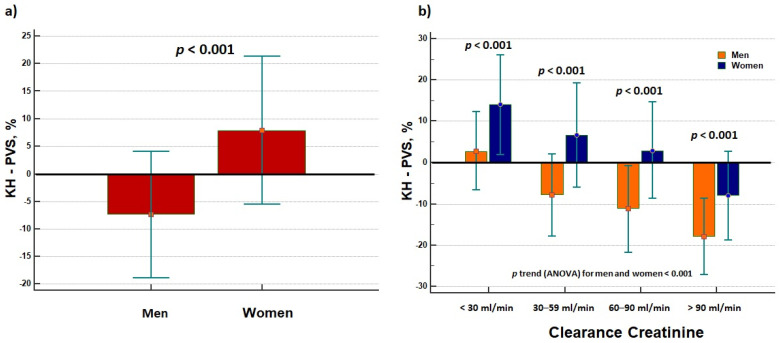
Plasma volume status (PVS) as calculated by means of Kaplan–Hakim (KH) formula: (**a**) differences between men and women, and (**b**) evaluation of creatinine clearance on the KH-PVS estimation between the two sexes.

In addition, KH-PVS was also higher in women than men and this effect was not related to renal function (Figure 3).

Both men and women with eGFR <30 mL/min showed increased values in KH-PVS, although the latter had the highest congestion status (Figure 3). Nevertheless, when eGFR was higher than 30 mL/min, men with AHF showed persistently reduced KH-PVS values while opposite results were detectable in women, as these demonstrated persistently increased KH-PVS values (Figure 3).

The multivariate logistic regression analysis demonstrated that KH-PVS, BUN/Cr, LVEF, and eGFR independently correlated with sex (Table 3).

Specifically, female sex increased the risk for higher KH-PVS and KM-PVS by 10%, while demonstrating a 6% increase in the risk of higher BUN/Cr ratio. Interestingly, the female sex was related to higher LVEF values (Table 3).

## 4. Discussion

The impact of congestion on the prognosis of patients with HF is well-established [9,10,22,32]. Indeed, the role of sex in the occurrence of congestion and evaluation of type of hyperhydration is still a matter of debate. Our study finally identified that: (1) in line with the literature, women more often suffered HFpEF, were older, and had device implantations less frequently than men; (2) clinical congestion markers—i.e., peripheral oedema, orthopnoea and jugular veins distention—did not specifically allow differentiating the congestion status between women and men, but BUN/Cr and PVS could effectively distinguish the congestion status in relation to sex; (3) PVS (as assessed by Duarte or Kaplam–Hakim formulas), BUN/Cr, LVEF, and eGFR independently correlated with sex.

Gerber et al. [5] reported a two-fold higher incidence in HFpEF in women than men, with a greater reduction over time (from 2000 to 2010) in the incidence of HFrEF. Data from the Framingham Heart Study and the Cardiovascular Health Study confirmed the increased prevalence in the incidence of HFpEF both in women and men, with the former trend incidence curve growing more steadily than the male one [33]. Stolfo et al. [7] pointed out that female patients with HF were more prone to be older, symptomatic, suffering from hypertension and kidney diseases, and less likely to undergo HF device therapy than men. Our study confirmed the results from the literature: women with AHF are more likely to suffer with HFpEF, to be older, and to have a reduced prevalence of pacemaker/implantable cardioverter defibrillator device therapy for their HF condition than men (Table 1).

We demonstrated no differences in terms of BNP plasma concentrations between the two groups. Although women seemed more susceptible to higher plasma concentrations of BNP [34], the literature showed controversial data about this biomarker between sexes [7,35]. Further specific studies are needed in order to better address such an issue.

Physical and clinical examination did not reveal any differences in terms of overt expressions of sign and symptoms of congestion between men and women. The rate of peripheral oedema, orthopnoea, and jugular venous distention were effectively not different between the two groups. Healthy women demonstrated reduced vasoconstrictive properties of their vascular tree, as well as smaller cardiac volumes and wall thickness after exercise training as compared to men [36]. Women are more prone to left and right ventricular-arterial uncoupling, and therefore higher left ventricle filling pressure and lower stroke volume, increased arterial stiffness and endothelial dysfunction, which predispose to increased pulmonary pressures and sex differences in pulmonary vascular reactivity, as well as poorer exercise tolerance [1,2]. These alterations in cardiovascular hemodynamics may account for the occurrence of signs and symptoms of HF. Shim et al. [37] demonstrated that women are more prone to develop arterial stiffness, and this was related to modification in the cardiac parameters of diastolic function. Espersen et al. [17] partially confirmed these characteristics: women with AHF showed similar prevalence in jugular venous distension and crackles on auscultation while demonstrating reduced prevalence in lower extremity oedema. Although no differences were according to instrumental evaluations (lung ultrasound and chest X-rays), the authors pointed out lower values in BUN and creatinine in women than men [17]. BUN and BUN/Cr ratio are reliable markers of neurohormonal activation and venous congestion [38]. Specifically, BUN/Cr seemed to play a prognostic role in patients with AHF [39,40,41], and may drive clinical diagnosis and therapy management. We demonstrated that women had higher BUN/Cr ratio values than men, thus demonstrating the higher congestion burden in women with AHF (Figure 1). This is the first study demonstrating a different burden of congestion between the two sexes. Such datum is corroborated by the analysis of the estimated PVS: women revealed higher values in PVS (Figure 1), independent from kidney function (Figure 3). Our analysis pointed out that women had a 25% higher risk of increased PVS than men, independent from the Duarte or KM formula adopted, and independent from confounding factors (Table 2). Nickander et al. [42] reported an increased blood volume and extracellular volume in healthy women as compared to men, independent from other confounding factors. Little data are available on AHF patients. Kobayashi et al. [43] retrospectively considered three cohort populations suffering from acute decompensated HF (ADHF) from the Tokyo Medical University hospital, the Centro Hospitalar do Porto, and Université de Lorraine (ICALOR study). Although univariate analysis reported that male sex was inversely associated to discharge-estimated plasma volume—as assessed by means of Duarte and Strauss formulas—in the Tokyo and ICALOR cohorts, the relationship was lost at multivariate analysis [44]. No mention was on female sex. Similar results were in Hudson et al.’s work [44]: male patients with ADHF did not differ according to changes in plasma volume tertiles. Indeed, Shirakabe et al. [45] demonstrated that male individuals with severe ADHF were more prone to show low PVS. Yoshihisa et al. [46] reported a higher prevalence of lower PVS in males than females with ADHF, this condition dramatically impacting on hard outcomes. While the subgroup analysis for all-cause mortality showed a statistically significant interaction *p*-value between genders, they effectively found a 2.4-fold increase in cardiac mortality and a 1.5-fold increase in cardiac event occurrence in female individuals with a higher increase in PVS [46]. While the prognostic impact of PVS in AHF has previously been demonstrated by our group [14,23], the present study demonstrated, for the first time, the role of female sex on the PVS in AHF patients. Men are less prone to develop increased PVS and venous congestion status as compared to women, but this datum was underestimated in the literature. As congestion status deeply impacts on the prognosis [10,47] and management [22,48] of HF patients, its exact evaluation and the main determinants of the fluid hemodynamics in HF are fundamental for the sake of individuals’ health and survival.

## 5. Conclusions

Sex influences the congestion status of individuals suffering from AHF. Specifically, the female sex was found to be directly related to PVS (evaluated by means of Duarte or Kaplam–Hakim formulas) BUN/Cr ratio, and venous and intravascular congestion biomarkers. A sex-guided approach to the correct evaluation of patients with AHF might become a cornerstone for the correct management of these patients.

## Figures and Tables

**Table 1:** Patient characteristics of the study population.

	Women (*n* = 252)	Men (*n* = 242)	*p* Level
**Clinical characteristics**			
Age, yrs	79 ± 9	77 ± 10	0.005
Weight, kg	69 ± 15	74 ± 13	<0.001
BMI, kg/mq	28 ± 6	27 ± 4	NS
NYHA functional class %			NS
II	6	9	
III	35	36	
IV	59	55	
**Physical examination**			
Jugular venous distention (>10 cm), %	59	55	NS
Peripheral oedema, %	53	45	NS
Orthopnoea, %	39	38	NS
**Medical history, %**			
Coronary artery disease	65	63	NS
Diabetes	29	26	NS
Atrial fibrillation	57	50	NS
PM/ICD	15	27	0.001
Prior HF	65	68	NS
**Laboratory values**			
LVEF,	45 ± 11	38 ± 11	<0.001
HFrEF, %	39	64	<0.001
HFmrEF, %	10	10	NS
HFpEF, %	51	26	<0.001
HFpEF, %	71	47	<0.001
Haemoglobin, g/dL	11.4 ± 1.9	12.6 ± 2.4	<0.001
Haematocrit, %	35 ± 6	38 ± 7	<0.01
Uric acid, mg/dL	7.4 ± 2.1	7.3 ± 2.1	NS
BUN, mg/dL	38 ± 20	36 ± 19	0.019
Creatinine, mg/dL	1.5 ± 1.0	1.5 ± 0.9	NS
eCrCl, mL/min per 1.73 m^2^	42 ± 25	47 ± 26	0.039
Sodium, mmol/L	139 ± 4	139 ± 4	NS
Potassium, mmol/L	3.9 ± 0.6	3.9 ± 0.6	NS
Chloride, mmol/L	101 ± 5	101 ± 6	NS
Serum osmolality, mOsm/kg	297 ± 21	294 ± 12	NS
Serum COP, mmHg	22 ± 3	22 ± 4	NS
Saturation O_2_, %	90 ± 6	90 ± 6	NS
PaO_2_, mmHg	62 ± 13	63 ± 12	NS
PaCO_2_, mmHg	43 ± 12	41 ± 11	NS
pH	7.43 ± 0.07	7.43 ± 0.07	NS
HCO_3_^−^, mmol/L	28 ± 6	27 ± 6	NS
**Home medications, %**			
Diuretic	83	82	NS
Beta-blockers	56	56	NS
ACE inhibitors/ ARBs	49	52	NS
MRAs	60	59	NS
Amiodarone	28	30	NS
Digitalis	20	19	NS
Ivabradine	5	7	NS
Calcium channel blockers	24	23	NS

**Abbreviations:** ACE: angiotensin-converting enzyme; ARB: angiotensin receptor blocker; COP: colloid osmotic pressure; BMI: body mass index; BNP: brain njatriuretic peptide; BUN: blood urea nitrogen; eCrCl: estimate creatinine clearance; HCO_3_^−^: standard bicarbonate; HF: heart failure; HFmrEF: heart failure with mildly reduced ejection fraction; HFpEF: heart failure with preserved ejection fraction; HFrEF: heart failure with reduced ejection fraction; ICD: implanted cardioverter/defibrillator; LVEF: left ventricular ejection fraction; MRAs mineralocorticoid receptor antagonists; NYHA: New York Heart Association; O_2_: oxygen; PaCO_2_: partial pressure of arterial carbon dioxide; PaO_2_: partial pressure of arterial oxygen; PM: pacemaker.

**Table 2:** Biomarkers of congestion according to sex and heart failure phenotipes.

	HFrEF	HFmrEF	HFpEF	*p* Level
**BNP**, pg/mL				
Women	1708 ± 1366 *	983 ± 874	1084 ± 977	<0.001
Men	2147 ± 1349 *	1441± 1236	1139 ± 960	<0.001
**Hydration, %**				
Women	78 ± 6.0	76 ± 3.6	79 ± 5.9	NS
Men	78 ± 5.7	80 ± 7.5	80 ± 7.2	NS
**BUN/Cr**				
Women	25 ± 9.3	24 ± 7.1	27 ± 8.9	NS
Men	23 ± 7.0	23 ± 9.3	22 ± 8.1	NS
**Duarte PVS,** dL/gr				
Women	6.0 ± 1.4	5.6 ± 1.1	6.1 ± 1.7	NS
Men	5.1 ± 1.5	5.4 ± 1.3	5.3 ± 1.6	NS
**Kaplan–Hakim PVS,** %				
**Women**	9.9 ± 14	3.5 ± 13	7.2 ± 13	NS
**Men**	−7.8 ± 11	−6.4 ± 12	−6.6 ± 12	NS
**Hydra Score**				
Women	3.2 ± 1.0	2.7 ± 1.5	2.9 ± 1.4	NS
Men	2.1 ± 1.3	2.4 ± 1.5	2.2 ± 1.4	NS

**Abbreviations: BNP:** Brain natriuretic peptide; **(BUN)/Cr:** blood urea nitrogen creatinine ratio, **HFmrEF:** heart failure with mildly reduced ejection fraction; **HFpEF**: heart failure with preserved ejection fraction; **HFpEF**: heart failure with reduced ejection fraction; **PVS:** plasma volumes status; * *p* < 0.001 vs. HFmrEF and HFpEF groups.

**Table 3:** Associations of age, left ventricle ejection fraction (LVEF), blood urea nitrogen (BUN)/creatinine (Cr) ratio, glomerular filtration rate (GFR), plasma volume status (PVS as estimated by Kaplan–Hakim’s [KH] formula) to female sex in logistic multivariate regression analysis.

Variables	Odds Ratio (95% CI)	*p*	*B* Coefficient	SE	Wald
Age, year	0.98 (0.96–1.01)	NS			
LVEF, %	1.05 (1.04–1.07)	<0.0001	0.055	0.009	31.3
BUN/Cr ratio	1.06 (1.03–1.09)	<0.0001	0.063	0.015	17.1
GFR, mL/min	0.98 (0.97–0.99)	=0.005	−0.013	0.004	7.9
KH-PVS, %	1.10 (1.08–1.13)	<0.0001	0.099	0.011	74.8

**CI:** confidential index; **SE:** standard error.

## Data Availability

Data will be available on request by contacting the corresponding author.
